# Nuclear translocation of TFE3 under hypoxia enhances the engraftment of human hematopoietic stem cells

**DOI:** 10.1038/s41375-022-01628-8

**Published:** 2022-06-22

**Authors:** Xuepeng Wang, Scott Cooper, Hal E. Broxmeyer, Reuben Kapur

**Affiliations:** 1 Department of Microbiology and Immunology, Indiana University School of Medicine, Indianapolis, IN 46202 USA; 2 Herman B Wells Center for Pediatric Research, Department of Pediatrics, Indiana University School of Medicine, Indianapolis, IN 46202 USA

**Keywords:** Haematopoietic stem cells, Haematological cancer

## To the Editor:

Hematopoietic stem cells (HSCs) reside in a hypoxic environment in vivo (~1–5% O_2_). When these cells are exposed to atmospheric levels of oxygen in ambient air (~21% O_2_), HSCs rapidly begin the process of differentiation and form hematopoietic progenitor cells (HPC) [1]. While majority of studies to date have analyzed HSC function(s) in ambient air, we have demonstrated that collection of cells in a hypoxia chamber (at 3% O_2_), not in ambient air, greatly increases HSC recovery from mouse bone marrow (BM), peripheral blood and human cord blood (CB) [1–3].

Transcription Factor binding to IGHM Enhancer 3 (TFE3) is a helix-loop-helix leucine-zipper transcription factor which acts as a master regulator of lysosomal biogenesis and immune response and plays a critical role in maintaining a state of pluripotency in mice and humans [4–6]. In naïve pluripotent state, TFE3 is located in the nucleus, but it resides in the cytosol in primed pluripotent cells where it regulates the exit from pluripotent state [6]. Although the function of TFE3 has been examined in regulating pluripotency, it is unclear whether this gene plays a role in hematopoiesis.

In order to collect human CD34^+^ cells under hypoxic conditions, we transplanted 1 × 10^6^ fresh CB CD34^+^ cells into sublethally irradiated immunodeficient NOD-SCID IL-2rγnull (NSG) mice. After 4 months of transplantation, human CD34^+^ cells were isolated from the bone morrow of recipient mice under hypoxic or ambient air conditions, respectively. The hypoxia collected CD34^+^ cells showed ~ 4-fold increase in the numbers of phenotypic Lin^−^ CD34^+^ CD38^−^ CD45RA^−^ CD90^+^ CD49f^+^ HSCs compared with cells harvested under ambient air conditions (Fig. S1A). In colony-forming unit (CFU) assay, ambient air collection of human CD34^+^ cells resulted in ~2-fold increase in the total number of granulocyte macrophage (CFU-GM) and multipotential (CFU-GEMM) colonies, relative to hypoxia collected cells (Fig. S1B).

**Fig. 1:**
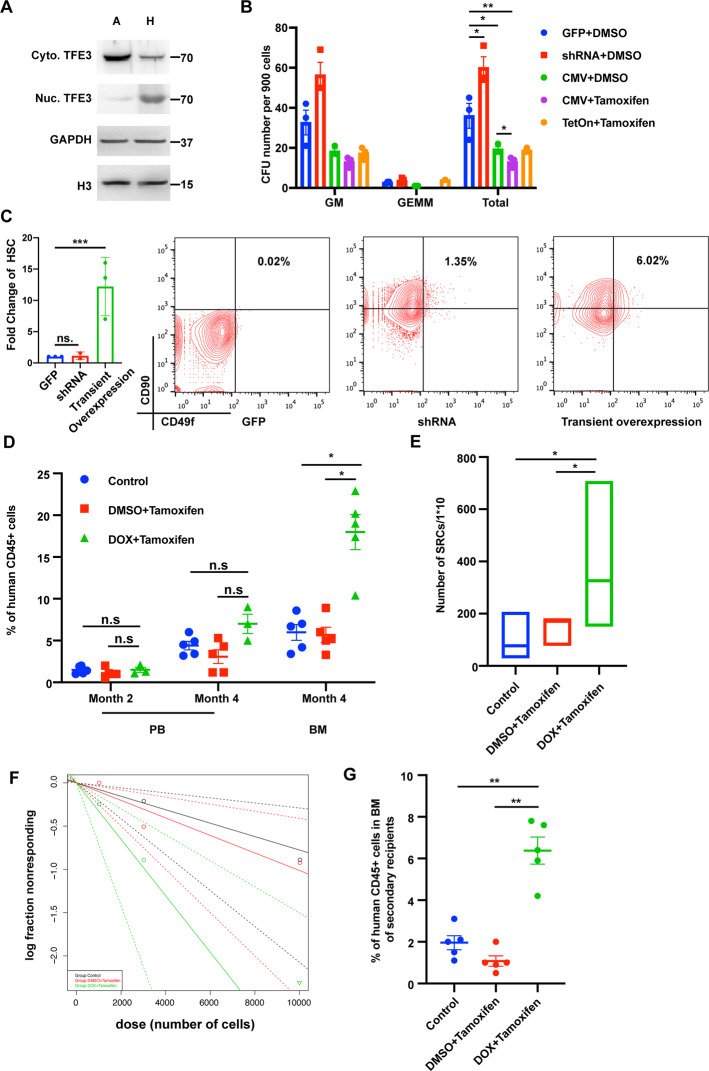
Forced nuclear overexpression of TFE3 in human CD34+ cells attenuates differentiation of HSCs to HPCs. **A** Western blot analysis of TFE3 expression profile in nuclear and cytoplasm extracts from ambient air and hypoxic condition collected human CD34^+^ cells. (A Ambient Air, H Hypoxic, Cyto.TFE3 Cytoplasm localized TFE3, Nuc. TFE3 Nuclear localized TFE3, GAPDH Control of cytoplasm protein, H3 Histone 3, Control of nuclear protein). **B** Colony output of GFP, shRNA, CMV-TFE3, TetOn-TFE3 transduced CB CD34+ cells. (*n* = 3). **E** Growth curve of sorted CD34^+^, GFP^+^ cells. (*n* = 3). **C** Left: fold change of HSCs in shRNA and TFE3 transiently overexpressed CB CD34^+^ cells. Right: representative flow cytometry plots (*n* = 3 independent experiments) showing phenotypic HSCs after TFE3 knockdown or transient overexpression in CB CD34^+^ cells. Gating was based on the use of isotype control antibodies. Percentages indicate gated cell populations among live cell events collected. (GFP + DMSO Cells transduced with GFP virus and treated with DMSO, CMV + DMSO Cells transduced with CMV-TFE3 virus and treated with DMSO, CMV + Tamoxifen Cells transduced with CMV-TFE3 virus and treated with tamoxifen, TetOn+ Tamoxifen Cells transduced with TetOn-TFE3 virus and treated with DOX and tamoxifen). **D** Percentage of human CD45^+^ cells in PB and BM at 2 months and 4 months after transplantation in NSG mice with 10,000 virus transduced cells (*n* = 5 mice per group). **E** HSC frequencies (line in the box) and confidence intervals (box) presented as numbers of SRCs in 1 × 10^6^ CB CD34^+^ cells. **F** Frequency of human SRCs in CB CD34^+^ cells. **G** Percentage of human CD45^+^ cells in BM at 4 months in secondary recipients. (Control GFP control, DMSO + Tamoxifen Transduced with TetOn-TFE3 virus and treated with Tamoxifen for 24 h, DOX + Tamoxifen Transduced with TetOn-TFE3 virus and treated with DOX and Tamoxifen for 24 h). Data shown as mean ± s.e.m. *p < 0.05; **p < 0.01; ***p < 0.001 by one-way ANOVA.

We have recently shown that HSCs collected in hypoxia show reduced activation of PI3Kinase and mTOR [3]. Given that mTOR is upstream of TEF3 [6], we explored the role of TFE3 by examining the cellular localization of TFE3 in human CD34^+^ cells. We performed western blot analysis on human CD34^+^ CB cells and found that when human CD34^+^ cells were collected under hypoxic conditions, most of the TFE3 protein was localized in the nucleus. In contrast, TFE3 was mostly localized in the cytosol of CD34^+^ cells collected in ambient air (Fig. 1A). Using real-time PCR, we analyzed TFE3 expression at RNA level in both CD34^+^ cells harvested under hypoxic or ambient air and found no significant difference in its expression under either condition (Fig. S1C). These results suggest that the collection methods used to harvest human CD34^+^ cells dramatically impact the TFE3 protein cellular localization but not its RNA expression. We next investigated the expression pattern of TFE3 in CB cells. We noted the expression of TFE3 mRNA in descending order from HSCs to HPCs, CMPs, GMPs and MEPs (Fig. S1D). Similar results were observed in mouse BM collected cells (Fig. S1E). Based on the western blot results and the expression pattern of TFE3 in hematopoietic cells, we hypothesized that TFE3 may play a role in regulating HSC differentiation.

A lentivirus-based system was used to knockdown or overexpress TFE3 in human CD34^+^ cells. Efficient downregulation of TFE3 expression was observed in shRNA transduced CD34^+^ cells (Fig. S1F). In order to force nuclear overexpression of TFE3 in CD34^+^ cells, we fused TFE3 coding region to the ERT2 domain. For constitutive or inducible induction of TFE3 expression, two types of overexpression systems were constructed using a CMV or a TetOn promoter, respectively. Real-time PCR analysis demonstrated that CD34^+^ GFP^+^ cells from the TEF3 overexpression group had a higher level of expression than control GFP^+^ cells (Fig. S1G). Furthermore, analysis of the TEF3 downstream target genes revealed a significant upregulation in their expression in CD34^+^ TEF3 overexpressing cells (Fig. S1H). Knockdown of TFE3 resulted in ~1.5-fold increase in total CFU-GM and CFU-GEMM colonies, relative to control cells, primarily due to a ~2-fold increase in CFU-GEMM colonies. In contrast, overexpression of TFE3 significantly decreased the number of total CFU colonies, especially in tamoxifen treated group (Fig. 1B). When TFE3 is forced to be expressed in the nucleus, the numbers of HPCs are dramatically reduced compared to control group. Furthermore, knockdown of TFE3 dramatically promotes cell expansion and long-term overexpression of TFE3 results in inhibition of cell proliferation (Fig. S1I).

Due to the observed inhibition of TFE3 overexpression on cell proliferation, we chose to transiently overexpress TFE3 by using the TetOn system. The virus transduced cells were treated with DOX and Tamoxifen for 24 h and then analyzed by flow cytometry. The percentage of phenotypic HSCs were dramatically increased by ~5-fold in TFE3 overexpressing cells compared to controls (Fig. 1C). Taken together, the knockdown and overexpression data suggest that TFE3 may play a role in the regulation of HSCs and HPCs in vitro. Overexpression increases the numbers of phenotypic HSCs but inhibits the activity of HPCs in vitro, while knockdown induces an opposite effect.

Phenotype of HSCs does not always recapitulate their functional activity [7]. Therefore, we assessed whether the engraftment efficiency of human CB CD34^+^ cells were enhanced by transient TFE3 overexpression. We performed limiting dilution assays (LDA) to assess numbers of functional HSCs and calculated numbers of SCID repopulating cells (SRC) in order to verify results of the in vitro HSC phenotype. TFE3 virus transduced fresh human CB CD34^+^ cells were treated with DOX and tamoxifen for 24 h and then GFP^+^ CD34^+^ cells were sorted by flow cytometry for transplantation. LDA results demonstrated that transient TFE3 overexpressed cells displayed enhanced engraftment relative to recipients of control cells in the BM at 4 months post-transplant (Fig. 1D). Compared with the control group, transient TFE3 overexpressing cells showed increased SRC frequency, a ~2-fold enhancement above that of the control group (Fig. 1E, F). Long-term reconstitution and self-renewal capability of transient TFE3 overexpressing CB CD34^+^ cells were confirmed by performing secondary transplantation. ~6-fold increase in chimerism was noted in transient TFE3 overexpressing CB CD34^+^ cells 4 months post-transplant (Fig. 1G). Thus, transient overexpression of TFE3 significantly enhances the engraftment of human CB CD34^+^ cells.

**Fig. 2:**
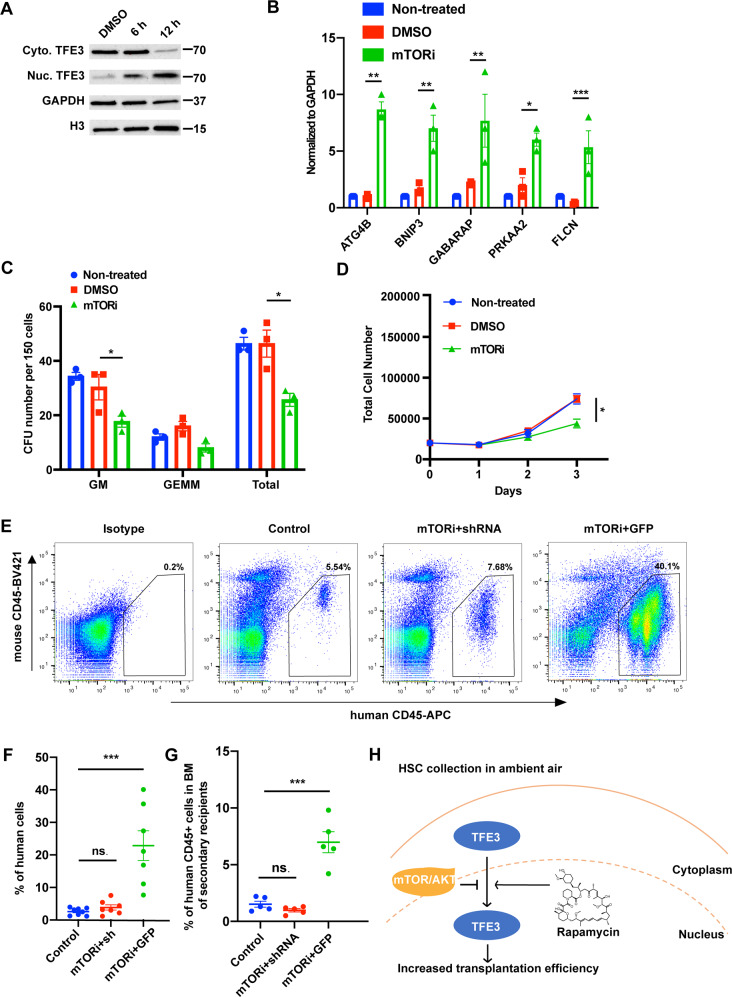
mTOR inhibitor induces TFE3 nuclear translocation and functionally mimics TFE3 overexpression. **A** Western blot analysis of TFE3 expression profile in nuclear and cytoplasm extracts from DMSO or mTORi treated CB CD34^+^ cells. (Cyto.TFE3 Cytoplasm localized TFE3, Nuc. TFE3 Nuclear localized TFE3, GAPDH Control of cytoplasm protein, H3 Histone 3, Control of nuclear protein). **B** Gene expression level of TFE3 targeted genes. (*n* = 3). **C** Colony output of non-treated, DMSO or mTORi treated CB CD34^+^ cells. (*n* = 3). **D** Growth curve of non-treated, DMSO or mTORi treated CB CD34^+^ cells. (*n* = 4). **E** Representative flow cytometer plots showing human CD45^+^, mouse CD45^−^ cells. Gating was based on use of isotype control antibodies. Percentages indicate gated cell populations among live cell events collected. **F** Percentage of human CD45^+^, mouse CD45^−^ cells in BM at 4 months. (Control GFP transduced cells, mTORi + sh mTORi treated and TFE3 shRNA transduced cells, mTORi + GFP mTORi treated and GFP transduced cells). **G** Percentage of human CD45^+^, mouse CD45^−^ cells in BM at 4 months in secondary recipients. **H** Model for the role of TFE3 in regulation of human CD34^+^ cells transplantation. TFE3 was mostly localized in the cytosol of CD34^+^ cells collected in ambient air. Induction of TFE3 nuclear localization in ambient air collected CB derived CD34^+^ cells mimics the nuclear expression of TFE3 under hypoxia and results in enhanced numbers of phenotypic and functional CB CD34 + HSCs. Data are shown as mean ± s.e.m. *p < 0.05; **p < 0.01; ***p < 0.001 by one-way ANOVA.

While transgene-based therapies can be associated with problems, small molecule-based methods may be safer and perhaps more convenient to implement. We thus explored whether we could find small molecules to induce TFE3 nuclear translocation in human CD34^+^ cells. In previous studies, mTOR inhibitors (mTORi) have been used to induce the nuclear translocation of TFE3 [6]. We therefore tested the FDA-approved mTORi, Rapamycin. After treatment of CD34^+^ cells with rapamycin for 12 h, nuclear located TFE3 was significantly enriched in human CB CD34^+^ cells (Fig. 2A). Real-time PCR showed a significant upregulation in the expression of TFE3 targeted genes in drug treated cells (Fig. 2B). Rapamycin treatment decreased total CFU numbers (Fig. 2C). In long-term culture of human CB CD34^+^ cells treated with Rapamycin, total cell number was significantly decreased (Fig. 2D). These results are consistent with our findings in cells overexpressing TFE3 in the nucleus. To confirm the regulatory network between mTORi and TFE3 and to further elucidate the function of mTORi treated human CB CD34^+^ cells, we transplanted shRNA transduced and rapamycin treated human CB CD34^+^ cells into NSG mice. An IPTG inducible shRNA system was used for transient knockdown of TFE3 in mTORi treated CB CD34+ cells. The flow cytometry results clearly demonstrate that the 12-h mTORi treatment significantly improved the engraftment efficiency of human CB CD34^+^ cells. Transient knockdown of TFE3 in the mTORi treated human CB CD34^+^ cells blocked this improvement (Fig. 2E). mTORi treated human CB CD34+ cells showed ~4-fold increase in the overall engraftment compared to controls (Fig. 2F). mTORi treatment did not induce changes in myeloid/lymphoid ratios of donor engrafting cells. (Fig. S1J). Long-term reconstitution and self-renewal capability of mTORi treated CB CD34^+^ cells were confirmed and a ~5-fold increase in chimerism was observed upon performing a secondary transplantation using these cells (Fig. 2G). These results suggest that mTORi treatment enhances human CB CD34^+^ cell engraftment by inducing the nuclear translocation of TFE3 (Fig. 2H).

The limited numbers of HSCs in a single unit of CB dominantly impacts the greater usage of CB for transplantation. Mitigation of oxygen shock through collection of CB in hypoxic chamber provides a way to acquire higher numbers of functional HSCs. In this study, we have shown that TFE3 nuclear localization contributes to the phenotype of hypoxia collected CB CD34^+^ cells. TFE3 specifically localizes in the nucleus of hypoxic collected CB CD34^+^ cells. Forced TFE3 nuclear expression by virus or Rapamycin treatment in CB CD34^+^ cells harvested and manipulated under ambient air, mimic the phenotype of hypoxia collected CD34^+^ CB cells. Greater numbers of HSCs and reduced numbers of more committed HPCs were observed in TFE3 nuclear overexpressed CB CD34^+^ cells. In vivo, after transplantation in NSG mice, these cells resulted in higher engraftment compared to control cells. These data provide mechanistic insights into TFE3 related networks and suggests that manipulation of such networks can improve the efficacy of HSC engraftment.

## Supplementary information

Supplementary Information

Supplementary Figure 1
