# Supplementary material for: Nuclear translocation of TFE3 under hypoxia enhances the engraftment of human hematopoietic stem cells
Source: Leukemia. 2022 Jun 22;36(8):2144–8. doi: 10.1038/s41375-022-01628-8 (PMC9343248; doi:10.1038/s41375-022-01628-8)
Supplement: Supplementary file 1 — Supplementary Information [file 41375_2022_1628_MOESM1_ESM.docx]

**Methods**

**Mice.** Immunodeficient 6- to 8-week-old NSG (NOD.Cg-Prkdcscid IL2rgtm1Wjl/ SzJ) mice were obtained from the In vivo Therapeutics Core at Indiana University School of Medicine (IUSM). The mice were maintained in the Laboratory Animal Resource Center (LARC) at IUSM. All animal experiments followed protocols approved by the Institutional Animal Care and Use Committee of IUSM.

**Human CD34^+^ CB cell collection and culture**. Umbilical CB units were supplied by Cleveland Cord Blood Bank and CordUse, Orlando, FL, USA. All studies were approved by the institutional review board of the IUSM. Mononuclear cells were isolated by density gradient centrifugation and CD34^+^ cells were isolated by immunomagnetic selection kit (Miltenyi Biotec, 130-046-702). The purity of human CD34^+^ CB cells was over 90% and the cells were cultured in StemSpanII serum-free medium (STEMCELL Technologies, 09650) supplemented with 100 ng/mL stem cell factor (SCF), 100 ng/mL FMS-like tyrosine kinase 3 ligand (Flt3L) and 100 ng/mL thrombopoietin (TPO). For mTOR inhibitor treatment, Rapamycin was ordered from Cayman Chemical (Ann Arbor, MI, USA). Briefly, Rapamycin (5μm) was used to treat the fresh purified CB CD34^+^ cells for 6 to 12 hours. For induction or knockdown of TFE3 expression, DOX (2ng/ml), Tamoxifen (100nM) or IPTG (100μm) were used to treat the virus transduced CD34^+^ cells for 24 hours and in vitro or in vivo assays were performed after the treatments.

**RNA extraction and real-time PCR**. After cells were harvested by FACS, RNA was extracted by a RNeasy Mini Kit following manufacturer’s protocol (Qiagen, 74106). Total RNA was reverse-transcribed by use of Superscript III kit (ThermoFisher, 18080093). Quantitative real-time PCR reactions were performed by SYBR Green PCR Master Mix (Thermo Fisher, Florence, KY, USA) and an Agilent Mx3000P QPCR System. Expression of housekeeping gene GAPDH was used as an internal control. Data are shown as relative mRNA level normalized to levels in vehicle control, set to 1.

**Vector construction and virus production.** TFE3 cDNA was cloned from CB CD34+ cell cDNA library. Briefly, fresh CB CD34^+^ cells were harvested and total RNA extracted. The total RNA was reverse-transcribed by use of Superscript III kit. TFE3 cDNA was ligated into an overexpression plasmid by use of In-Fusion HD Cloning Plus kit (Takara, 638920). For shRNA vector construction, single-strand oligos were synthesized by Genewiz. After annealing, double-strand oligos were ligated into the shRNA expression plasmid by T4 ligase (NEB, M2622L). Well-constructed vectors were sent to Genewiz (South Plainfield, NJ, USA) for sequencing. For inducible induction of TFE3 knockdown, well-constructed vectors were ordered from Millipore, Sigma (St. Louis, MO, United States).

**Lentivirus transduction of CB CD34^+^ cells.** Lentiviruses were concentrated by 30% percutaneous endoscopic gastrostomy 8000 (PEG8000, Sigma, 1546605). Before transduction, freshly isolated CB CD34^+^ cells were cultured in stemspanII medium with SCF, Flt3L and TPO for 6 hours, 20μm CsH was added to the medium and cells were cultured for 16 hours. Lentivirus was added in the same medium at a MOI of 50–200 and incubated for 8 hours, a step which was repeated 3 times. Cells were then cultured in stemspanII medium with SCF, Flt3L and TPO allowed to recover for two days before in vitro or in vivo assays were performed.

**Flow cytometry and sorting.** Fresh or cultured CB cells were sorted for different phenotypes by using the following antibodies. Mouse anti-human antibodies were used to detect CD34 (APC, BD Biosciences, 560940), CD45RA (Taxe-red, BD Biosciences, 562298), CD90 (BV421, BD Biosciences, 562556), CD49f (cy5.5, BD Biosciences, 562495), CD38 (PE, BD Biosciences, 560981), Lin (FITC, Biolegend, 348807) ，Lin (FITC, BD Biosciences, 340546). CD3 (BV421, BD Biosciences, 562427), CD33 (PE, BD Biosciences, 555450), CD19 (PE, BD Biosciences, 555413) and CD45 (APC, BD Biosciences, 555485). Cells were analyzed on a BD FACS-Canto flow cytometer (BD Biosciences), and cell sorting (under low sheath fluid pressure) was conducted on a BD FACSAria cell sorter (BD Biosciences, Franklin Lakes, NJ, USA).

**Colony-forming unit (CFU) assay.** GFP^+^,CD34^+^ cells harvested after cell sorting by FACS at day 8 were plated in semi-solid methylcellulose culture medium in presence of 30% Fetal bovine serum (FBS) (GE Healthcare, HyClone, SH30071.03), 2 mM l-glutamine (Lonza, 17-605E), 100 μM β-mercaptoethanol (Sigma, M6250), 1 U/mL erythropoietin (EPO) (R&D Systems, 287-TC-500), 50 ng/mL SCF (R&D Systems, 7466-SC-010/CF), 10 ng/mL IL-3 (R&D Systems, 203-IL-050/CF) and 10 ng/mL GM-CSF (R&D Systems, 7954-GM-010) and were cultured at lower (5%) O_2_ and at 5% CO_2_ in a humidified incubator. Number of CFU-GM- and CFU-GEMM-colonies were scored with an inverted microscope 14 days after culture in semi-solid medium. This culture medium allows detection of CFU-GM- and CFU-GEMM- colonies, but not BFU-E colonies.

**Limiting dilution analysis (LDA).** Frequency of human SCID repopulating cells (SRCs) was determined by LDA as reported before. Increasing doses of GFP overexpressing CD34^+^ cells (2500, 5000 or 10000 cells) were intravenously injected into sublethally irradiated NSG recipient mice (350 cGy; 137Cs source, single dose). Four months after transplantation, the percentage of GFP+ human CD45+ cell chimerism was analyzed by immunostaining and flow cytometry. For long-term engraftment assays that assess the self-renewal capacity of HSC, 3 × 10^6^ BM cells from primary recipients of the 10000-cell group were intravenously transplanted into secondary sublethally irradiated NSG recipient mice.

**Statistical analysis.** Statistical analysis was performed by use of Microsoft Excel and GraphPad Prism (GraphPad Software, San Diego, CA, USA). Data are shown as mean ± s.e.m. (as indicated in the figure legends). One-way ANOVA was used to compare differences in means between more than two groups, as indicated.

**Supplementary Figure Legends**

**Supplementary Figure 1**. **Hypoxic collection and processing of human CD34+ cells alter TFE3 protein localization. A**. Fold change of HSCs in ambient air and hypoxic condition collected human CD34+ cells. (A: Ambient Air. H: Hypoxic). **B.** Colony output of ambient air and hypoxic condition collected human CD34+ cells. (n = 3 cultures from 2 experiments). **C.** TFE3 expression level in ambient air and hypoxic condition collected human CD34+ cells. (n=3). **D.** TFE3 expression level (n = 4 independent pooled CB samples). TFE3 expression across the human hematopoietic hierarchy. **E.** TFE3 expression level in mouse hematopoietic hierarchy. (n=3 bone morrow from independent mice). **F.** Schematic of the lentivirus used to knockdown TFE3 and control GFP. **G.** Schematic of the lentivirus used to overexpress TFE3 and control GFP. **H.** Gene expression level of TFE3 targeted genes. (n=4). **I.** Growth curve of sorted CD34+, GFP+ cells. (n = 3). J. Myelo-lymphopoiesis in recipients in month 4. Data shown as mean ± s.e.m. *P < 0.05; **P < 0.01; ***P < 0.001 by one-way ANOVA.
