# Supplementary figures and images for: Nuclear translocation of TFE3 under hypoxia enhances the engraftment of human hematopoietic stem cells
Source: Leukemia. 2022 Jun 22;36(8):2144–8. doi: 10.1038/s41375-022-01628-8 (PMC9343248; doi:10.1038/s41375-022-01628-8)

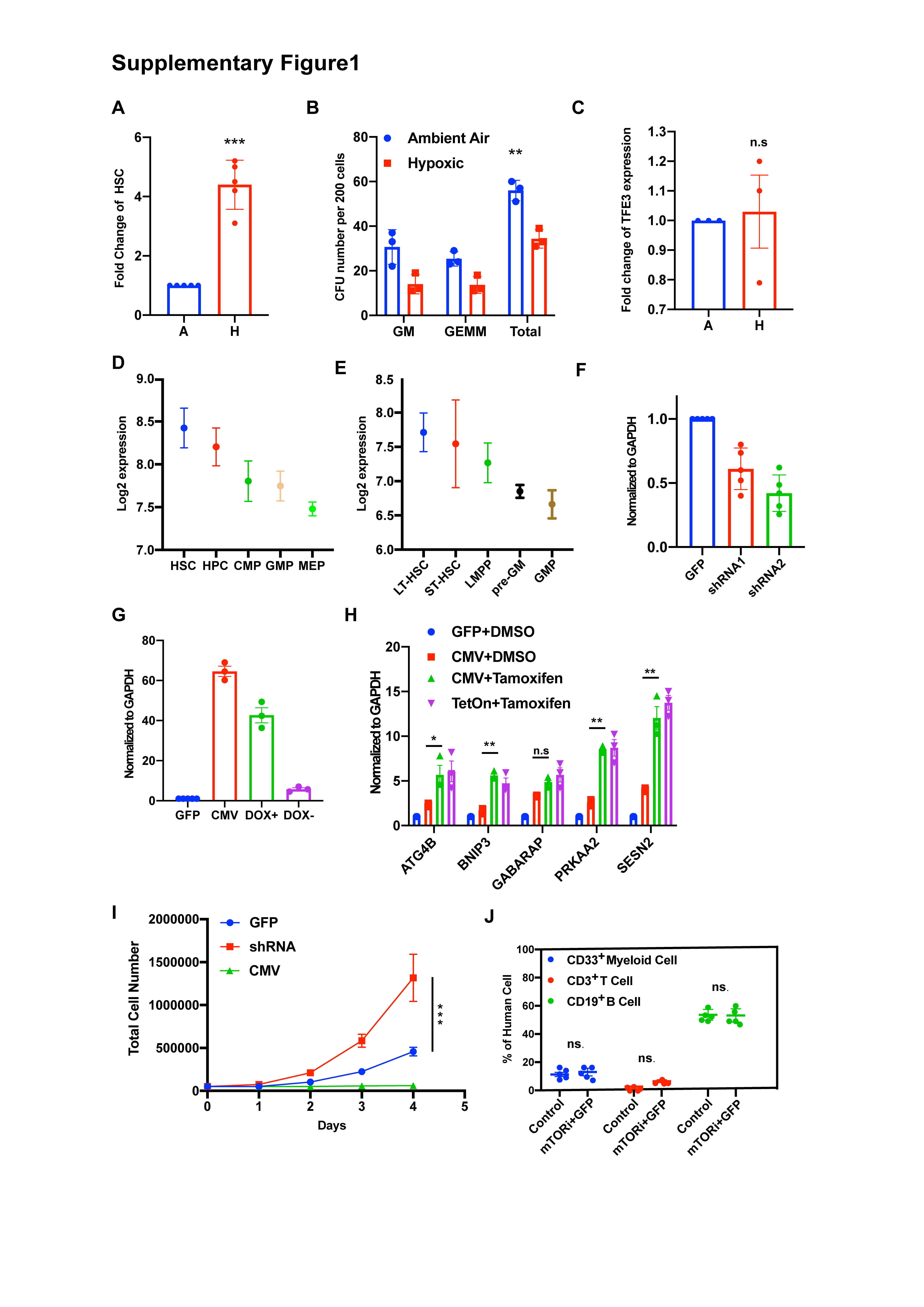

Supplement: Supplementary file 2 — Supplementary Figure 1 [file 41375_2022_1628_MOESM2_ESM.tif]
